# Sonographic Detection of Surgical Site Fluid Collections and Postoperative Maternal Morbidity Following Cesarean Section

**DOI:** 10.7759/cureus.36836

**Published:** 2023-03-28

**Authors:** Aruna Verma, Neelu Shrivastava, Garima Sharma, Aditya Sharma

**Affiliations:** 1 Obstetrics and Gynecology, Lala Lajpat Rai Memorial Medical College, Meerut, IND; 2 Preventive Medicine, Government Medical College, Badaun, IND

**Keywords:** postoperative morbidity, ultrasonography, cesarean section, surgical site infections, surgical site fluid collection

## Abstract

Introduction: Surgical site infection (SSI) is one of the most common complications after cesarean and causes much burden on the mother as well as the health care system. SSIs are defined as infections of a surgical site up to 30 days after surgery. Ultrasonography of the surgical site may be a helpful tool to detect its complication. With this background, the following study was planned to evaluate the clinical significance of sonographically detected fluid collections and post-operative maternal morbidity following cesarean section (CS) and identify risk factors associated with their formation.

Methods: This prospective observational study was conducted at the Department of Obstetrics and Gynecology, Lala Lajpat Rai Memorial Medical College, Meerut. A total of 1000 women, who had undergone CS were included. Sonographic examination of the cesarean site was done on the 3^rd^ or 4^th^ postoperative day to look for any fluid collection in the parities or pelvis. All cases were followed on the 8^th^ postoperative day and finally on the 30^th^ postoperative day to look for any SSIs i.e. surgical wound problems like wound infection, induration, and discharge from a surgical wound, or even wound dehiscence and postoperative morbidity.

Results: Out of the total cases (1000), abdominal wound fluid collection was noted in 490 (49%) women after CS. Thirty-two patients were lost to follow-up, so 458 patients were followed, of which collection was septated or loculated in 62 (13.6%) and diffused in 396 (86.5%). Out of 62 loculated and 396 diffused cases, 21 (33.87%) and 20 (5.05%) cases reported surgical site abdominal wound infection and needed resuturing, respectively and it was found to be highly significant (p<0.001).

Conclusion: Post-operative fluid collections are common after CS. But it is the pattern of the fluid collection that determines post-operative wound infection and morbidity. Thus ultrasound of the cesarean site may be an important tool to detect surgical site wound complications earlier and to decrease postoperative morbidity.

## Introduction

Cesarean section (CS) is a major life-saving obstetric intervention that protects both mother and fetus from pregnancy and childbirth-related complications. As per the latest National Family Health Survey (NFHS-5 2019-2020) data, the CS rate in India is around 21.5% [1]. The overall post-cesarean complication rate is 14.5%, and the most common complication is infection (13.3%). These infections include endometritis, wound infection, infected hematoma, and abscess formation, with endometritis being the most common (6.6%), followed by urinary tract infection (3.1%) and wound infection (1.6%). Compared to emergency CSs, elective CSs reportedly have a lower complication rate [2].

Various high-risk pregnancies like preeclampsia, eclampsia, increasing maternal age, previous abdominal surgeries other than CS, gestational diabetes mellitus, and multiple pregnancies, have been documented as risk factors for CS-related complications. Maternal obesity (body mass index (BMI) > 35 kg/mm2) use of corticosteroids for any medical disorder and diabetes mellitus has also been found to be highly associated with wound complications. Labor-related risk factors that predispose patients to postoperative morbidity include prolonged rupture of membranes, increased duration of labor before surgery, and anemia; typical symptoms that prompt imaging in the immediate postoperative period include fever, a dropping hemoglobin level, unexpected heavy vaginal bleeding, and pain [3].

Surgical site infections (SSIs) are defined as infections occurring up to 30 days after surgery (or up to one year after surgery in patients receiving implants) and affecting either the incision or deep tissues at the operation site [4]. Almost half of the women have sonographically detectable fluid collection following CS; however, few prospective studies have been conducted to evaluate the association between sonographically detected fluid collection and postoperative morbidity. The most common site for fluid collection after CS is the abdominal wall (range, 3-150 mL), and this is referred to as parietal wall collection which is defined as any subcutaneous or subfascial echo-free area that is seen on ultrasound as a fixed, hypoechoic, and heterogeneous area. Small peritoneal fluid collections, such as an anterior sub-facial hematoma and a bladder flap hematoma, are generally considered routine and are usually not significant if less than 4 cm in size [5].

In addition to SSIs, postoperative febrile morbidity is also common after CS. Therefore, the present study was an attempt to determine abdominal surgical site fluid collection by ultrasound and its association with post-cesarean maternal morbidity among all the patients who underwent CS between January 2021 and June 2022 at the Department of Obstetrics and Gynecology at Lala Lajpat Rai Memorial Medical College and associated Sardar Vallabh Bhai Patel Hospital, Meerut.

## Materials and methods

This prospective observational study was conducted at the Department of Obstetrics and Gynecology from January 2021 to June 2022 at the Lala Lajpat Rai Memorial Medical College and associated Sardar Vallabh Bhai Patel Hospital, Meerut. The study protocol was approved by the Institutional Ethics Committee of Lala Lajpat Rai Memorial Medical College, Meerut (No./SC-1/2022/7690).

A total of 1000 women who had undergone CS and were willing to participate were included in the study. This was after the study was explained to them and they had given written and informed consent.

The exclusion criteria included patients receiving anticoagulants other than those for thrombophlebitis prophylaxis; human immunodeficiency virus (HIV), hepatitis C virus (HCV), or hepatitis B surface antigen (HBsAg) reactive cases, and subjects who had declined consent. Women were included consecutively, except when the participating sonographers were unavailable.

Detailed demographic parameters, complaints at admission, and history (obstetric, gynecologic, past medical and surgical, personal, and family) were recorded. For selected women, all labor details, examination findings (general physical, systemic, and detailed obstetric), the indication for surgery, the surgical technique, the duration of the procedure, and the amount of blood loss were also recorded.

Postoperative morbidity including fever, blood loss, and serous fluid discharge from the incision, was noted. Axillary temperature was measured every eight hours, and febrile morbidity was defined as a temperature of at least 37.5◦C on any two in-hospital days after surgery excluding the first 24 hours. We chose a relatively low cut-off value (at least 37.5◦C twice, excluding the first postoperative day) to increase the sensitivity of the detection of fever. Also, in our study, all women who had undergone CS received prophylactic antibiotics as per the protocol at our hospital.

All enrolled women had undergone a sonographic examination on the third or fourth postoperative day to assess for the presence of parietal or pelvic fluid collection. The sonographic evaluation was performed by sociologists who were blinded to the women’s clinical history before examination and were not involved in any clinical decision-making.

**Figure 1:**
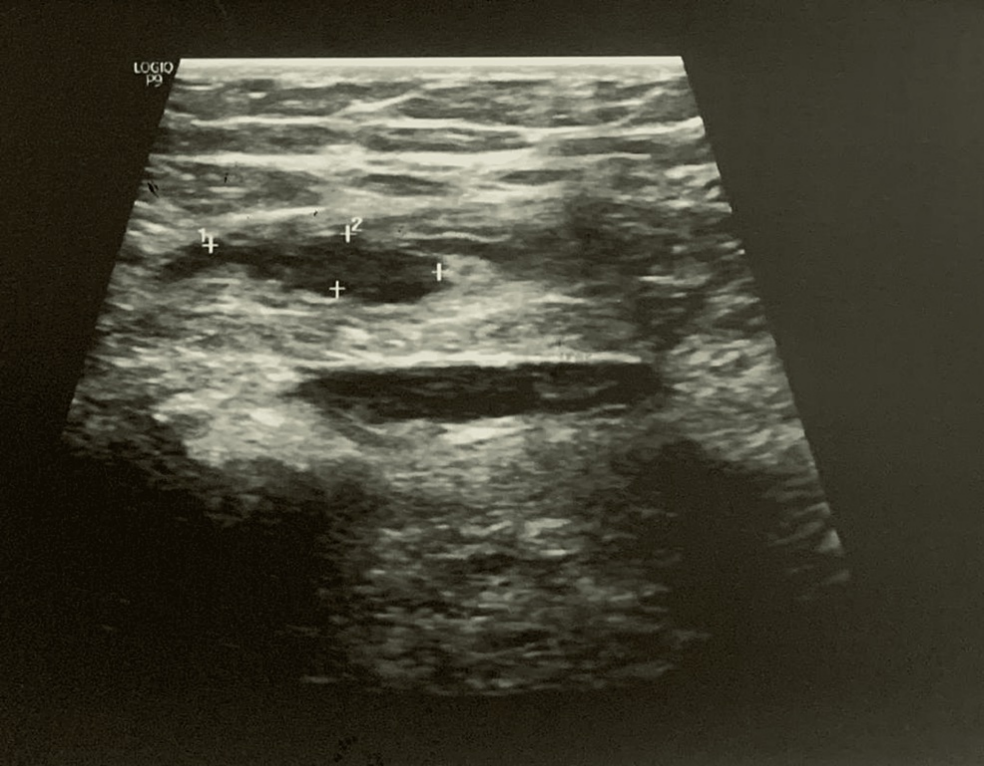
Minimal abdominal wound collection

**Figure 2:**
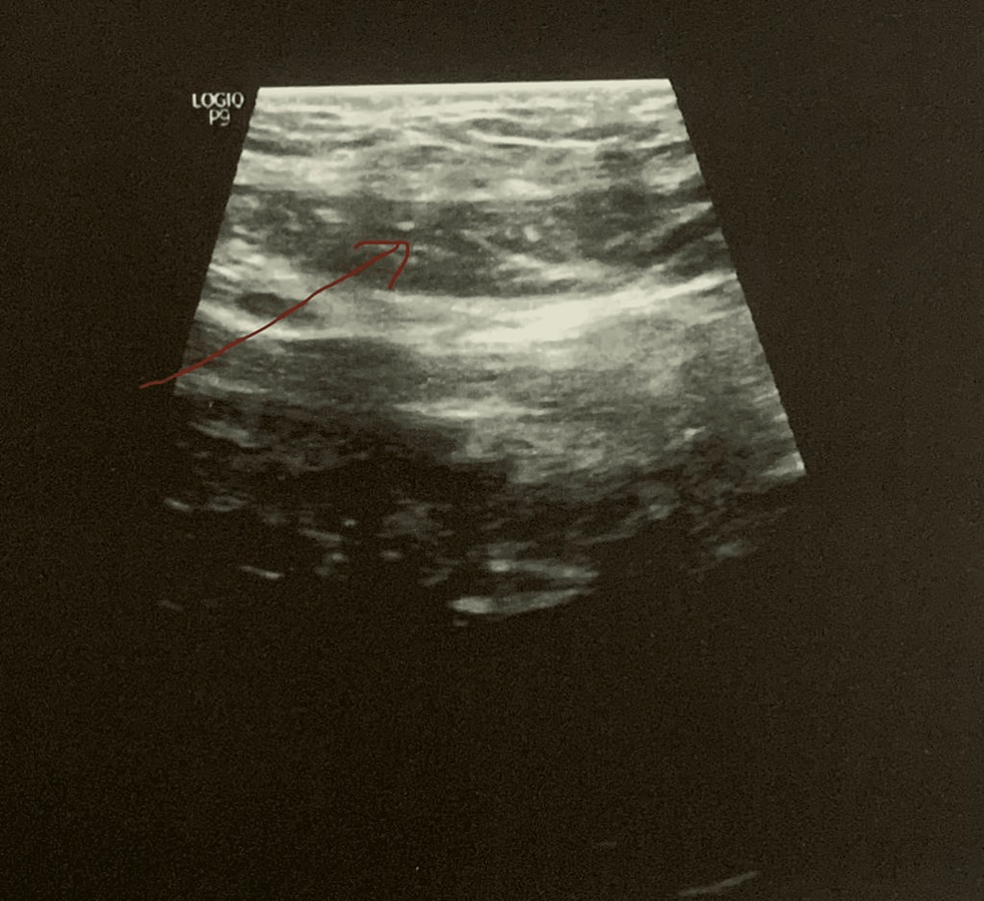
Diffuse abdominal wound collection

**Figure 3:**
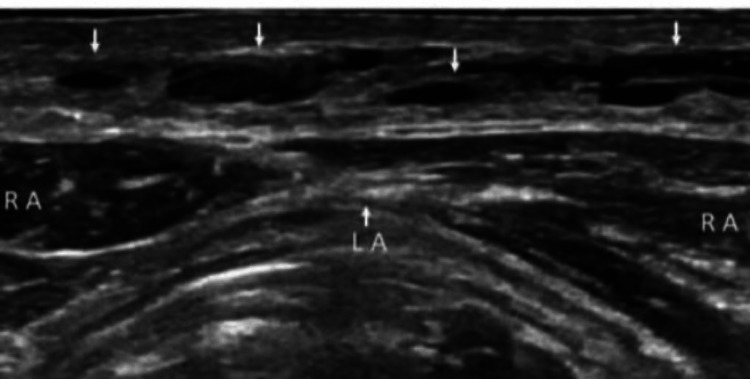
Loculated abdominal wound collection

**Figure 4:**
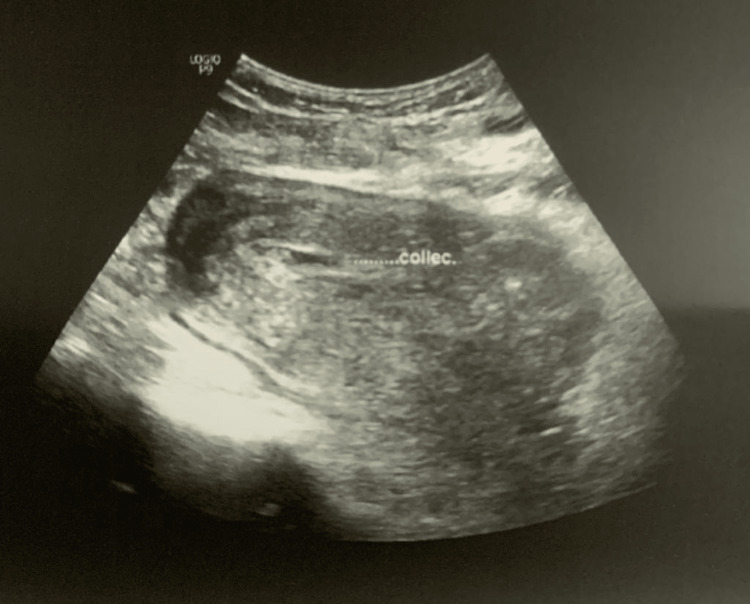
Minimal intrauterine collection

The examination was performed using a real-time ultrasound scanner with either a 3.5 or 6 MHz abdominal probe and was conducted with the patients in the recumbent position. The three main diameters of any detected echo-free areas were measured, and the radius was obtained by dividing this measurement by two. The volume of the fluid collection was calculated using the formula for an ellipse (4/3* x r1 x r2 x r3). The endometrial cavity, the bladder flap area below the rectus abdominis muscle, and the abdominal wall were systematically examined. Characteristics of the fluid collection were recorded which might be minimal, diffuse, or loculated (Figures 1-3). Intrauterine collection was also noted, if any (Figure 4).

The participants were followed up on the fourth day post lower-segment CS (LSCS), then on the seventh or eighth day (day of stitch removal), and finally on the 30th day following LSCS. The condition of the stitch line and other complaints were evaluated, and all details were recorded on the working proforma. Management of patients was not compromised due to study planning and was done as per management guidelines.

Statistical significance was assessed with the chi-square or Fisher exact test in the case of proportions and with Mann Whitney-U and independent paired-t tests in the case of continuous variables. A p-value < 0.05 was considered statistically significant. Data analysis was performed using SPSS (IBM Corp., Armonk, NY).

## Results

**Figure 5:**
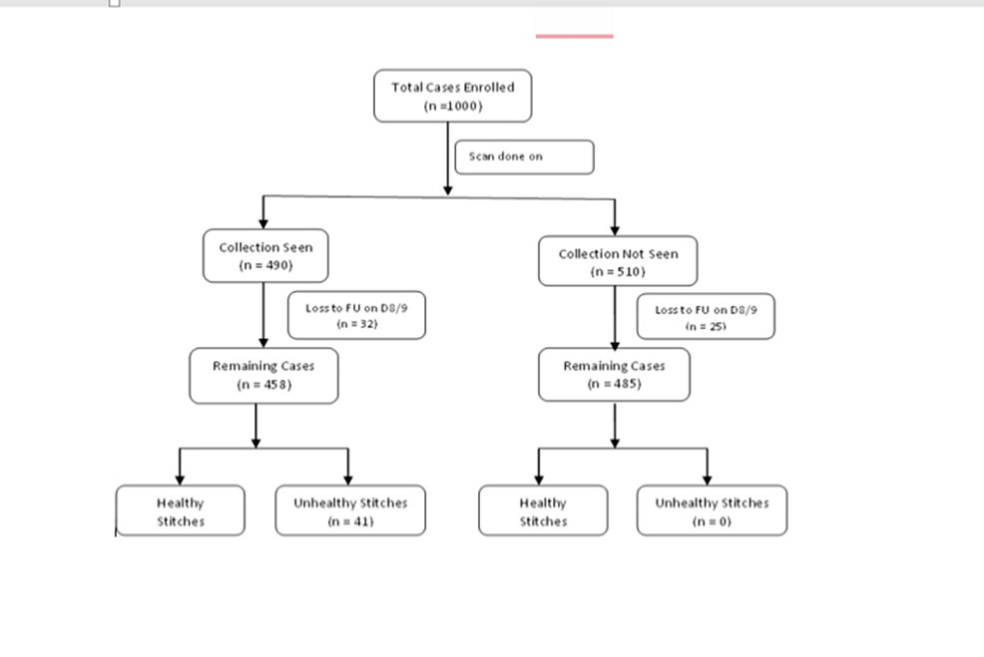
Study flow

During the study period, a total of 1000 participants were enrolled as per our inclusion and exclusion criteria. Three or four days after CS (depending on the patient’s comfort level), a sonographic evaluation of the surgical site was performed. Of the 1000 participants, 510 had no significant collection in the stitch line, and 25 of them did not come for follow-up, so only 485 from this group were followed up to postoperative day 30. Similarly, out of the other 490 participants who had a significant collection in the stitch line, 32 were lost to follow-up, and only 458 were followed up to postoperative day 30 (Figure 5).

The mean age of the total 1000 participants was 25.25 (±2.81) years, and BMI was 29.65±2.14. Mothers with greater age (25.70±2.91 years) were not associated with the development of fluid collection compared to mothers with lesser age (24.84±2.65 years). Duration of labour was also significantly associated with the development of fluid collection (p=<0.05). One hundred and forty-nine mothers with ruptured membranes, 15 mothers with pre-operative fever, and 48 mothers with post-operative fever were significantly associated with the development of the fluid collection. In our study, the most common indication was a previous cesarean with scar tenderness. Uterine closure was done in a continuous non-locking fashion in maximum cases, however, results were statistically insignificant and had no role in the formation of collection at the operating site. A significant association was found with a duration of cesarean, peritoneal closure, and the antibiotic given. However, in cases where the uterine angle got extended and repaired, fluid collection in the stitch line was seen and results were statistically significant (Table 1).

**Table 1:** Distribution of cases (n=1000) according to the presence of abdominal stitch line fluid collection SD: standard deviation, CS: cesarean section, LSCS: lower segment cesarean section, Hb: hemoglobin

PARAMETERS		Collection present (n=490)	Collection absent (n=510)	p-value
Age (years, mean SD)		25.70±2.91	24.83±2.65	0.099
Body Mass Index (mean SD)		29.63±1.83	29.69±2.41	1.89
Primiparous (n%)		163(33.2%)	187(36.6%)	0.054
Multiparous (n%)		327(66.7%)	323(63.1%)	0.047
Duration of labor (mean SD-in hours)		4.612±1.889	3.963±1.112	<0.033
Status of membranes before caesarean section (n%)	Intact	341(69.5%)	295 (57.8%)	0.039
	Ruptured	149 (30.4%)	215 (42.1%)	0.021
Phase of labor (n%)	Active	75 (15.3%)	80(15.6%)	1.798
	Latent	415 (84.6%)	430(84.3%)	1.008
Indication of CS (n%)	Previous 1 CS	167(34.1%)	167(32.7%)	0.044
	Previous 2 CS	70(14.3%)	59(11.5%)	0.050
	Previous 3 CS	4 (0.8%)	2 (0.2%)	0.048
	2^nd ^stage arrest	3(0.6%)	1(0.1%)	0..02
	Other	246( 50.2%)	286( 55.5%)	0.168
Blood Pressure >140/90 (n%)		16(3.3%)	11(2.2%)	0.004
Hemoglobin (gm/dl) (mean SD)	6-8	6.8±0.57	6.16±0.24	0.088
	8-11	9.83±0.84	9.82±0.8	1.112
	>11	11.95±0.6	12.48±0.88	0.037
Pre operative fever (n%)		15(0.3%)	6(1.1%)	0.012
Previous CS with adhesion in parity (n%)		48(9.8%)	46((.0%)	0.005
Non closure of peritoneum		352(71.8%)	284(55.7%)	0.017
Uterine angle extension		16(3.3%)	0	<0.01
Type of uterine suture	Continuous locking	50	55	0.48
	Continuous non locking	440	455	
Duration of CS (>45 minutes)		438(89.4)	239(57.4)	0.003
Post operative fever (n%)		48(9.8)	9(0.1)	0.045
Duration of antibiotics	2 days	377(76.9)%)	375 (73.5%)	0.055
	5 days	113(23.1%)	135 (26.5%)	0.039

After patient follow-up on days 4-8 (excluding those who were lost to follow-up, n=25), we observed that in the collection absent group, all stitch lines were healthy, while in 41 of the 458 mothers in the collection present group, an unhealthy stitch line was observed (excluding those lost to follow-up, n=32 ). Table 2 depicts the association of the possible risk factors with the development of an unhealthy stitch line. Higher maternal BMI (30.16±1.30 kg/m2) was more significantly associated with the development of an unhealthy stitch line compared to a lesser maternal BMI (29.56±1.86 kg/m2). Duration of LSCS and labor was also significantly associated with the development of an unhealthy stitch line (median duration of 61.52±16.85 minutes and 4.42±4.196 hours, respectively, in the healthy stitch line group and 69.52±16.07 minutes and 7.634±7.644 hours, respectively, in the unhealthy stitch line group).

In contrast to patients who had no maternal anemia (n=190), no parity adhesions (n=427), no peritoneal closure (n=401), and no post-operative fever (n=410), anemia (n=268), adhesions in parity (n=31), and post-operative fever (n=48) were significantly associated with the development of an unhealthy stitch line (Table 2).

**Table 2:** Distribution of cases (n=458) according to the condition of stitch line among patients who had sonographically detected collection in the stitch line SD: standard deviation, BMI: body mass index, CS: cesarean section, LSCS: lower segment cesarean section, BP: blood pressure, Hb: hemoglobin

PARAMETERS		Healthy stitch line (n=417) (91.0%)	Unhealthy stitch line (n=41) (9.0%)	p-value
AGE (years, mean SD)		25.62±2.859	26.51±3.264	0.059
BMI (mean SD)		29.572±1.885	30.17±1.316	0.011
Primiparous (n%)		143 (31.2%)	8 (1.7%)	0.081
Multiparous (n%)		274 (59.8%)	33(7.2%)	0.081
Duration of labour (in hours)		4.423±4.196	7.634±7.644	0.0002
Phase of labor (n%)	Latent	350 (76.4%)	38 (8.3%)	0.208
	Active	67 (14.6%)	3(0.7%)	0.0002
Indication of CS (n%)	Previous 1 LSCS	143 (31.2%)	11 (2.4%)	0.230
	Previous 2 LSCS	51(11.1%)	10 (2.2%)	0.230
	Previous 3 LSCS	4 (0.9)	0 (0%)	0.230
	2nd stage arrest	22(4.8%)	3(0.7%)	0.230
	others	197(43.0%)	17(3.7%)	0.230
BP >140/90 (n%)	Present	24(5.2%)	4(0.9%)	0.301
	Absent	393(85.8%)	37(8.1%)	
Hb (mean SD)	No anaemia	174(38%)	16(3.5%)	<0.0003
	Mild anaemia	108(23.6%)	1(0.2%)	
	Moderate anaemia	135(29.5%)	24(5.2%)	
	Severe anaemia	0(0)		
Pre operative fever (n%)	Present	34 (8.1%)	14 (34%)	0.0003
	Absent	383(91%)	27(65%)	
No. of CS with adhesion in parity (n%)	Present	22(4.8%)	9(2%)	0.001
	Absent	395(86.2%)	32(7%)	
Non closure of peritoneum	Present	44(9.6%)	13 (2.8%)	0.002
	Absent	373(81.4%)	28(6.1%)	
Uterine angle extension	Present	15	1	1.00
	absent	402	40	
Type of uterine suture	Continuous locking	29	5	0.213
	Continuous non locking	388	36	
Duration of LSCS ≥45min		61.52±16.85	69.88±16.07	0.002
Post operative fever (n%)	Absent	3x83(91%)	27(65%)	0.684
	Present	34 (8%)	14 (34%)	
Duration of antibiotics	2 days	318(69.4%)	32 (7%)	0.796
	5 days	99 (21.6%)	9(2.0%)	0.796
Status of membrane	Intact	287(62.7%)	24(5.2%)	0.178
	Ruptured	130(28.4%)	17(3.7%)	

In this study, 27.8% of participants had a minimal intrauterine collection and 15.2% had a mild intrauterine collection. Most of these participants (86.5%) had diffused type fluid collection in their abdominal stitch line while 13.6% had loculated type fluid collection. Out of the total cases, minimal to <5cc collection was noted in 722 (72.2%) cases (minimal collection means unmeasurable according to the sonologist, therefore it was ignored). Measurable collection up to 5 cc was seen only in 331 cases. These results were statistically significant (p= <0.001), showing that the stitch line became unhealthy with an increasing amount of collection and as the type of collection changed from diffuse to loculated (Tables 3-4).

**Table 3:** Distribution of cases (n=458) according to the amount of fluid collection ml- mililitre

Amount of fluid collection	Healthy stitch line (n=417)	Unhealthy stitch line (n=41)	P value= <0.001 (Via fisher exact)
<5ml	329(71.8%)	2(0.4%)	
5-10ml	84(18.3%)	13(2.8%)	
10-20ml	4(0.9%)	20(4.4%)	
>20ml	0(0%)	6(1.3%)	

**Table 4:** Distribution of cases (n=458) according to the type of fluid collection

Type of fluid collection	Healthy stitch line (n= 417)	Unhealthy stitch line (n=41)	P value= <0.001 (Via chi square)
Diffuse (396) 86.5%	376(94.95%)	20(5.05%)	
Loculated (62) 13.6%	41(66.13%)	21(33.87%)	

On the third or fourth postoperative day, most (80.8%) patients were asymptomatic; however, 9.6% of them had tenderness along the stitch line, 5% had an induration in the stitch line, 10.1% had discharge from the stitch line, and 12.9% had seroma and hematoma around the stitch line. These results were statistically significant (p=<0.001), showing their association with unhealthy stitch line. Only 2.6% of patients with an unhealthy stitch line had uterine tenderness, and this was not statistically significant (Table 5).

**Table 5:** Distribution of cases (n=458) according to factors leading to post-cesarean morbidity D3/D4: postoperative day 3/day 4

Parameters		Healthy stitch line (n=417)	Unhealthy stitch line (n=41)	
Seroma/ Hematoma	Present	34(7.4%)	25(5.5%)	<0.001
	Absent	383(83.6%)	16(3.5%)	
Tenderness in Stitch line	Present	32(7.0%)	12(2.6%)	0.0001
	Absent	385(84.1%)	29(6.3%)	
Uterine Tenderness	Present	11(2.4%)	01(0.2%)	1.0
	Absent	406(88.6%)	40(8.7%)	
Induration on D3/D4	Present	11(2.4%)	12(2.6%)	0.001
	Absent	406(88.6%)	29(6.3%)	
Discharge from Stitch line	Present	32(7%)	14(3.1%)	0.0001
	Absent	382(84%)	27(5.9%)	

In maximum cases (58.8%), stitch removal was done on the eighth day and the rest were done on the ninth (27%) or another postoperative day. Most (95%) women had a healthy stitch line on the day of stitch removal, while 5% of patients had an unhealthy stitch line. Of these 41 patients, 34 had a gapped stitch line on the day of stitch removal, while nine reported on postoperative day 30 with an unhealthy/gapped stitch line; all of them needed resuturing. Two of the patients who reported a gapped stitch line on the day of stitch removal and were resutured again reported with a gapped stitch line on follow-up day 30 and needed resuturing. Most of the women whose stitch line gapped had a collection of ≥20 cc in their stitch line, and the nature of fluid collection was septated in most of them.

## Discussion

The present study was planned to study the surgical site fluid collection by ultrasound with post-cesarean maternal morbidity. The study was approved by the ethical committee of our institute and written consent was taken from the patient or his/her relatives. This prospective study was carried out with the enrolment of 1000 confirmed cases, to analyse the surgical site fluid collection by ultrasound with post-cesarean surgical site complications in the form of SSIs, wound dehiscence, etc.

In our study, 57 out of the 1000 participants were lost to follow-up; therefore, postoperative day 30 follow-up could not be done. Most of the mothers (96.9%) were aged between 21 and 30 years, with the mean age being 25.29 years. Socio-demographic factors were found to be insignificant in our study. Similar to our study, Antonelli et al. (2004) reported a mean age of 29.6 years in their study participants [3]. Morbid obesity has a dramatic effect on pregnancy outcomes. In our study, the mean BMI was 29.62±2.13 kg/m2, and a BMI of >30 kg/m2 was found to be significantly associated with an unhealthy stitch line.

We found that a prolonged duration of labor with a median of four hours was significantly associated with the development of an unhealthy stitch line (p=0.002). In our study, rupture of membranes prior to CS was significantly associated with fluid collection in the stitch line but not with an unhealthy stitch line. Furthermore, an unhealthy stitch line was found to be significantly associated with CS done in the active phase of labor. In our study, many patients (28.4%) had moderate anemia, and this was significantly associated with the development of fluid collection in the stitch line (p=0.001) as well as with an unhealthy stitch line (p<0.0003). Similar to our study, Regmi et al. (2022) revealed that out of 1135 CSs, 97 developed SSIs, with an incidence rate of 8.54%. Multiple risk factors like age, obesity, medical complications during pregnancy, labor status during CS, prolonged duration of rupture of membranes (for more than 18 hours), and more than five vaginal examinations before the procedure increased the chances of SSIs following CS [6]

After comparing intraoperative factors, we found that adhesions in parities, continuous locking or non-locking of uterine sutures, and parietal peritoneum non-closure do not affect the stitch line, while prolonged LSCS was found to be associated with an unhealthy stitch line (p= <0.002) and surgeries with uterine angle extension were reported to be associated with fluid collection at the stitch line (p<0.001). However, Naeiji et al. (2021) found that there was no statistically significant relationship between the duration of the surgery and the volume of free fluid four and 24 hours after CS [7]. Turan et al. (2014) found that the unlocked uterine closure technique is safe and does less damage to the myometrium [8].

During the sonographic assessment of the collection at the surgical site, the amount and type of collection were also evaluated. We found that 27.8% had a minimal intrauterine collection and 15.2% had mild intrauterine collection; most women (86.5%) had collections in the abdominal stitch line, while no collection was seen in pelvic parities. We also found that stitch line outcomes became poor with an increase in the amount of fluid collection. Additionally, outcomes were affected by the type of collection, with the loculated type of collection having poorer outcomes than the diffuse type. Antonelli et al. [3] found that following CS, the fluid collection was present in 69 (48%) women; it was located in the abdominal wall in 58 of them and in the pelvis in 11. No woman had collections on both sites. Naeiji et al. (2021) found that four hours after CS, minimal, moderate, and large amounts of free fluid were seen in 38 (38%), 45 (45%), and 17 (17%) patients, respectively [7].

CS rate has increased dramatically during the last decade, decreasing perinatal morbidity and mortality; however, it is also associated with an increased incidence of maternal postpartum morbidity, particularly infections and febrile morbidity. In our study, we found that patients with the fluid collection have a statistically significant association with seroma/hematoma formation, tenderness, induration, and discharge from the stitch line, which further predisposes them to an unhealthy stitch line and wound dehiscence. This establishes the association of fluid collection in the abdominal wall with stitch line infection in postpartum morbidity. However, febrile morbidity was not significantly associated with an unhealthy stitch line. Similarly, Antonelli et al. (2004) found that the occurrence of postoperative fever with no obvious cause is common. However, they did not demonstrate any association between postoperative fever and fluid collection detected by ultrasound [3]. In contrast, Toglia and Pearlman reported an association between pelvic fluid collection and febrile morbidity [9].

Several studies have shown that ultrasound findings should be interpreted according to the patient’s clinical features and should not be used as a single diagnostic modality to predict outcomes [10-11]. Machado et al. (2012) found that morbidly obese women with a BMI >40 kg/m2 are at increased risk of pregnancy complications and a significantly increased risk of requiring cesarean delivery [11]. These studies also emphasize that the free fluid as well as the echogenicity in the uterine cavity after delivery (vaginal or cesarean) may be associated with retained placental bits [12-13] and may have no clinical importance; such patients will often remain asymptomatic [14-16].

## Conclusions

Postoperative fluid collections are common following CS. Collection may be anywhere in the surgical site like in the uterus, in the pelvis, or the abdominal wall. Primarily, the type and amount of fluid collection determine the wound complications. Therefore, surgical wound assessment by sonography is a useful and cost-effective adjunct that the obstetrician can use to predict surgical site complications and wound dehiscence.
